# The summating potential polarity encodes the ear health condition

**DOI:** 10.1007/s00018-023-04809-5

**Published:** 2023-05-24

**Authors:** Pierre Hakizimana

**Affiliations:** Department of Biomedical and Clinical Sciences (BKV), Linköping University, 581 83 Linköping, Sweden

**Keywords:** Cochlear potentials, Hair cell receptor activation, Noise-induced hearing loss, Basolateral K^+^ channels, Operating point shift, Hearing damage mechanisms

## Abstract

The summating potential (SP), the DC potential which, along with the AC response, is produced when the hair cells convert the vibrational mechanical energy of sound into electrical signals, is the most enigmatic of the cochlear potentials because its polarity and function have remained elusive for more than seven decades. Despite the tremendous socioeconomic consequences of noise-induced hearing loss and the profound physiological importance of understanding how loud noise exposure impairs the hair cell receptor activation, the relationship between the SP and noise-induced hearing impairment remains poorly characterized. Here, I show that in normally hearing ears, the SP polarity is positive and its amplitude relative to the AC response grows exponentially across frequencies, and becomes negative and decreases exponentially across frequencies following noise-induced hearing injury. Since the SP is thought to be generated by K^+^ outflow down the gradient through the hair cell basolateral K^+^ channels, the SP polarity switch to negative values is consistent with a noise-induced shift in the operating point of the hair cells.

**Supplementary Information:**

The online version contains supplementary material available at 10.1007/s00018-023-04809-5.

## Introduction

The cochlear hair cells have an extraordinary ability to convert wide ranges of sound frequencies and intensities into receptor potentials [1–10], which are ultimately processed in the brain to produce the hearing sensation. Exposure to loud sounds compromises hair cell receptor activation, thereby impairing hearing. Recently, it has been discovered that the widespread use of personal listening devices at unsafe levels puts more than 1 billion teenagers and young adults at risk of hearing loss [11]. However, the mechanisms underlying noise-induced receptor dysfunction remain poorly understood. To help increase awareness about the dangers posed by unsafe listening habits, a better understanding of how noise-induced hearing loss develops is necessary.

Receptor potentials are produced when the vibrational mechanical energy associated with sound causes K^+^ and Ca^2+^ ions to permeate the mechanoelectrical transduction (MET) channels, resulting in sinusoidal voltage oscillations around the membrane potential of the hair cells (reviewed in [12]). The tonotopic organization of ion channels, including the MET channels, contributes to the electrical response properties and frequency tuning of hair cells [12]. While the MET channels in inner hair cells (IHCs) have a relatively uniform MET and K^+^ conductance across the cochlea [5, 8, 13, 14], those in outer hair cells (OHCs) have a conductance that varies with frequency, with a gradded ~ fivefold increase from the apex to the base of the cochlea [15, 16]. K^+^ conductances in IHCs include those with slow (G_Ks_) and fast (G_Kf_) activation kinetics, and a small amount of delayed rectifier K^+^ current (G_Kn_), which are also tonotopically organized and contribute to frequency tuning [13, 17, 18]. OHCs mainly express a voltage-dependent K^+^ channel composed of KCNQ4 subunits (G_Kn_) [19], activated at unusually negative membrane potentials [15], and large-conductance Ca^2+^-activated K^+^ channels (BK channels), which regulate OHC electromotility and sound signal amplification, and are also tonotopically organized [15].

Receptor potentials generated by the MET channels consist of the AC and DC components. The AC component encodes the frequency and amplitude of the sound stimulus [1–8, 8–10], while the exact polarity and role of the DC response, or the SP, remain poorly understood despite extensive study over the past seven decades. SP can be recorded intracellularly or extracellularly [20–26], with the polarity varying among studies in humans and animal models. Recently, Fettiplace [12] highlighted a study that showed that when an outward K^+^ current passes through the basolateral membrane of isolated IHCs, G_Kf_ and G_Ks_ generate positive DC waveforms resembling SPs [17], suggesting that the outward movement of K^+^ ions through the basolateral K^+^ channels produces the SP waveform [12]. This is consistent with the idea that the small resting open probability of the MET channels in the IHCs should rectify the voltage excursion much more on the positive half of the sinusoidal response than on the negative half of it. In contrast, the OHCs with a resting open probability of 50% [15, 27] should have no such positive voltage rectification, causing the response to remain sinusoidal with increasing stimulus intensity [15]. Consequently, the SP component of extracellular recordings should predominantly come from the IHCs, while the AC component should predominantly come from the OHCs [15]. In other words, the SP should be predominantly controlled by the operating point of the IHCs.

Therefore, under normal conditions, the SP in normally functioning ears should have a positive polarity, while a negative polarity would indicate a pathological condition. This study aims to test these ideas and investigate whether a negative SP can characterize noise-induced hearing loss in guinea pig temporal bone preparations [28–30]. Unlike in vivo studies, the present study did not detect a neural component; therefore, this component is not included in the analysis.

## Results

The temporal bone preparation from the guinea pig [28–30] was deemed suitable for addressing the issues above for the following reasons. A literature survey led to a paper published in Nature in the 1970s, in which it was postulated that the unusual variability in polarity and magnitude of the SP could be explained by difficulties in controlling the physiological condition of the specimen during electrophysiological recordings [22]. A possible source for such instability could be tissue oxygenation, because subjecting guinea pigs to a brief period of hypoxia induced a surprising but reversible 79% decrease in the SP amplitude [31]. However, tissue oxygenation is not an issue in the ear temporal bone preparation from the guinea pig because oxygen is easily dissolved in the perfusion cell culture medium [28, 29] (see ref [32] for a picture of the preparation showing how it is connected to the perfusion tube). Moreover, confocal imaging of the hearing organ in vivo showed that the native hair cell morphology is well preserved in the temporal bone preparation [33] and its sound-evoked electrical responses (see below) are generally consistent with those recorded in vivo in terms of amplitude and nonlinear dependency in sound intensity [34]. In addition, we recently used a modified version of this preparation (albeit with a larger apical cochlear opening than usual) to investigate low frequency encoding by the hearing organ and the results were consistent with those we obtained in unopened cochlea in *vivo* [35].

**Fig. 1:**
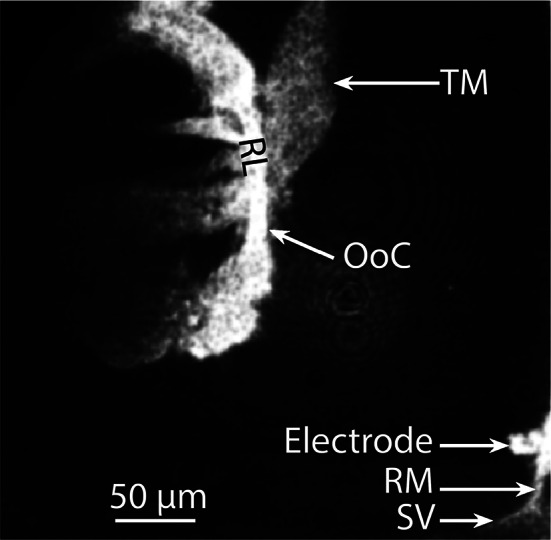
Reflection confocal image highlighting the location of the recording electrode relative to the organ of Corti (OoC) in the guinea pig cochlear apical region (best frequency ~ 180 Hz). *RM* Reissner’s membrane, *RL* reticular lamina, *TM* tectorial membrane, *SV* stria vascularis

A key feature of the guinea pig temporal bone preparation is that it has a small opening in the bone of the cochlear apical region [28, 36], which is soft and shallow, thus easily amenable for a gentle and non-traumatic dissection. The small apical opening is then used to insert, via a computer-controlled micromanipulator, an endolymph-filled glass micro-electrode into the endolymphatic space (the cochlear fluid in which the hair cell stereocilia bathe) through an intact Reissner’s membrane under video monitoring by confocal microscopy (see Methods). It is this electrode that was used to extracellularly record the sound-evoked electrical potentials of the hair cells (see Fig. 1 for the position of the recording electrode relative to the organ of Corti) reported in the present paper.

### The summating potential in normally hearing ears is positive across frequencies

To investigate the mechanisms underlying SP production in the hair cells, sound-evoked electrical potentials were recorded in the temporal bone preparation described above at 20 different frequencies evenly distributed between 60 and 820 Hz and at 8 different sound intensities ranging from 41 to 77 dB SPL. This yielded a dataset of 160 sound-evoked electrical recordings, each corresponding to a unique combination of sound frequency and intensity. These recorded responses were carefully analysed in MATLAB to characterize the AC and SP waveforms across frequencies and sound intensities in different conditions.

**Fig. 2:**
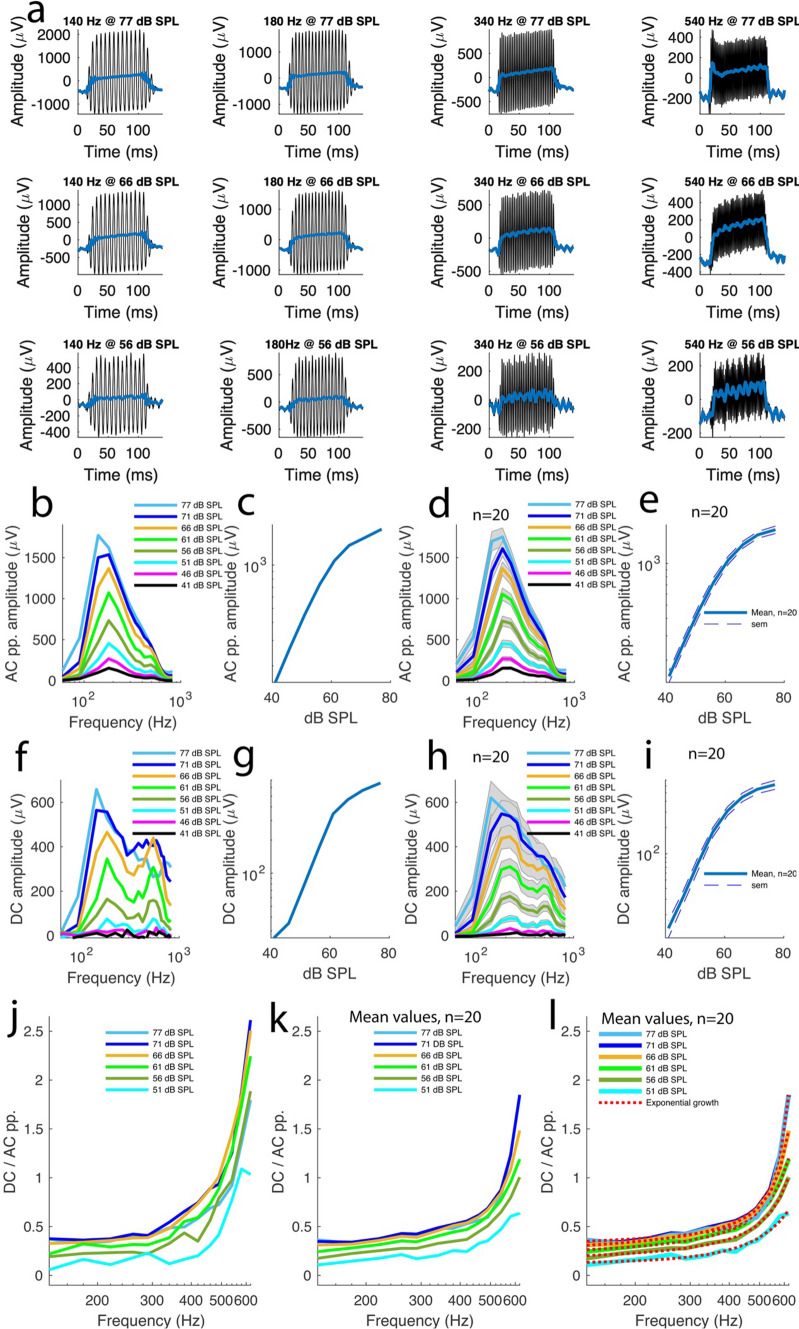
The summating potential in a normally hearing ear is positive across frequencies. **A** 12 of 160 waveforms of sound-evoked electrical potentials from a representative control preparation. Each recording is a mean of 10 different acquisitions. The sound frequency and intensity are displayed at the top of each recording. Blue lines were added to the plots to make it easier to visualize the summating potential (SP) waveforms. However, the actual SP amplitudes were computed from the raw data, not the blue lines, as detailed in the main text. **B** Frequency-tuning of AC peak-to-peak amplitude for the preparation shown in (**A)**. **C** Sound intensity dependency of the peak amplitudes for the tuning curves in (**B)**. **D** Averaged frequency-tuning curves for the AC peak-to-peak amplitudes across 20 preparations from 20 different animals. Shaded plots depict s.e.m. **E** Peak amplitude values from the tuning curves in D plotted against sound intensity. **F** SP amplitude frequency-tuning curves for the preparation shown in (**A)**. **G** The peak amplitudes for the tuning curves in F plotted against sound intensity. **H** Averaged SP amplitude frequency-tuning curves across 20 preparations from 20 animals. Shaded plots depict s.e.m. **I** SP peak amplitudes from the tuning curves in H plotted against sound intensity. **J** Ratios between SP amplitude and AC peak-to-peak amplitude plotted against frequency for different sound intensities for the preparation shown in (**A**). **K** Averaged ratios between SP amplitude and AC peak-to-peak amplitude plotted against frequencies at different sound intensities across 20 preparations from 20 animals. **L**. The averaged plots in K were fitted with a double exponential function f(x) = a*e^(b*x)^ + c*e^(d*x)^. See supplemental Table 1 for the fitting parameter values

Figure 2a shows 12 of the 160 recordings described above in the same preparation in control conditions.

To characterize the AC response behaviour across frequencies and sound intensities, the 160 recordings were subjected to Fourier analysis in MATLAB and the peak-to-peak amplitudes extracted and quantified. The frequency-tuning curves for the preparation described above are shown in Fig. 2b. The peak-to-peak amplitude was frequency-tuned for each sound intensity tested (Fig. 2b). The peak amplitudes were 1772 µV at 77 dB SPL; 1537 µV at 71 dB SPL; 1368 µV at 66 dB SPL; 1071 µV at 61 dB SPL; 733 µV at 56 dB SPL; 455 µV at 51 dB SPL; 268 µV at 46 dB SPL and 154 µV at 41 dB SPL. A nonlinear relationship was evident when these amplitudes were plotted against sound intensity (Fig. 2c).

These features were consistent across all 20 preparations. The mean peak-to-peak amplitudes were frequency-tuned across all the sound intensities tested (Fig. 2d) and the peak amplitudes from these tuning curves were sound intensity-dependent nonlinearly. The averaged peak amplitude values were 1752 ± 107 µV at 77 dB SPL; 1609 ± 90 µV at 71 dB SPL; 1368 ± 78 µV at 66 dB SPL; 1056 ± 72 µV at 61 dB SPL; 727 ± 59 µV at 56 dB SPL; 453 ± 42 µV at 51 dB SPL; 268 ± 25 µV at 46 dB SPL and 153 ± 14 µV at 41 dB SPL. As shown by Fig. 2e, these amplitudes exhibited a nonlinear relationship versus sound intensity with a slope of 49 ± 3 µV/dB SPL (Fig. 2e; mean ± s.e.m., n = 20 independent preparations from 20 different animals).

To investigate the SP behaviour across frequencies and intensities, the SP waveforms were systematically analysed in the 160 sound-evoked electrical recordings above. As shown by Fig. 2a for 12 of the 160 same-preparation recordings (blue traces), the SP waveforms were generally frequency and intensity sensitive. From these recordings, it is also evident that each SP waveform is characterized by 5 distinct steps (Fig. 2a). The first step (step 1) corresponds to a baseline lasting 14 ms and is followed by a rapidly rising step that lasts 8 ms (step 2). Then follows a much longer, steadier but slowly rising step that lasts 85 ms (step 3) followed by a rapidly declining step lasting 8 ms (step 4) and the SP waveform ends with another baseline lasting 25 ms (step 5). To compute the SP amplitude from the raw data in each recording, the mean value for the baseline region of the recording (in the raw data) was subtracted from the mean value for the region of the recording corresponding to step 3 (in the raw data) above. These SP amplitudes were then plotted as function of frequency for different sound intensities.

As shown by the tuning curves for the example preparation in Fig. 2a, the SP amplitude was frequency-tuned at the different sound intensities tested, with peak amplitudes of 658 µV at 77 dB SPL; 564 µV at 71 dB SPL; 465 µV at 66 dB SPL; 345 µV at 61 dB SPL; 164 µV at 56 dB SPL; 75 µV at 51 dB SPL; 35 µV at 46 dB SPL and 26 µV at 41 dB SPL (Fig. 2f). Plotting these peak amplitudes against sound intensity revealed a nonlinear relationship (Fig. 2g). Across 20 preparations, the features above persisted. As shown by Fig. 2h, the mean SP amplitude values were frequency-tuned. The max mean amplitude values were 620 ± 74 µV at 77 dB SPL; 548 ± 61 µV at 71 dB SPL; 446 ± 52 µV at 66 dB SPL; 311 ± 36 µV at 61 dB SPL; 177 ± 22 µV at 56 dB SPL; 78 ± 14 µV at 51 dB SPL; 32 ± 6 µV at 46 dB SPL and 14 ± 3 µV at 41 dB SPL. Plotting these values against sound intensities confirmed a nonlinear relationship with a slope of 19 ± 2 µV/dB SPL (Fig. 2i, n = 20 preparations from 20 guinea pigs).

The mean tuning curves made it clear that the SP amplitudes were highly frequency tuned, especially at 77 and 71 dB SPL (Fig. 2h). Between 66 and 51 dB SPL the peaks broadened progressively and at 46–41 dB SPL became nearly flat. Such a dependency of the SP amplitude tuning in sound intensity is not apparent in the AC tuning curves (Fig. 2d), supporting that both potentials are driven by different mechanisms.

The SP tuning curves also made it clear that the SP amplitudes were positive across frequencies and sound intensities. Such a stability of the SP positive polarity across frequencies and SPLs contrasts with the SP polarity instability reported by previous studies [22, 23, 25, 26].

Remarkably, the data described above are in agreement with an earlier prediction that the physiological SP polarity is positive due to the fact that K^+^ ions entering the cell through the MET channels are expelled from the cell down the gradient by the basolateral membrane K^+^ channels [17]. Since the K^+^ channel populations involved can directly be inferred from the shape of the SP waveform [12, 17], it is apparent that the low frequency SP is produced by G_Kf_ because the waveforms did not contain the slowly adapting component thought to be present at high frequencies [12]. In addition, the SP waveforms from the present study were similar with the DC waveforms obtained when a depolarizing current of 131 pA was injected through the hair cell basolateral membrane after the slow K^+^ channels (G_Ks_) type were blocked [17]. However, at least one waveform containing fast and slow components is seen in Fig. 2a at 77 dB SPL and 540 Hz. Although this is rather exceptional, it indicates that both G_Kf_ and G_Ks_ contribute to the SP in the low frequency cochlear region but that the G_Ks_ contribution to the SP may become more important at higher frequencies.

To further characterize the SP behaviour, the SP-to-AC amplitude ratios were computed across frequencies and sound intensities. To that end, a couple of considerations were made. Since the AC response amplitudes were highly tuned (Fig. 2b and d), values outside the peak regions logically fell quickly towards the baseline and therefore the 140–620 Hz frequency band was used for this analysis. Likewise, the sound intensities of 41 and 46 dB SPL yielded response amplitudes close to the baseline (Fig. 2g and h) and therefore the amplitude values corresponding to these two sound intensities were not included in the ratio analysis.

In the example preparation shown in Fig. 2a, the SP-to-AC amplitude ratios increased with frequency and this trend was maintained across the sound intensities tested (Fig. 2j). Across 20 preparations from 20 different guinea pigs, these features persisted. At 77 dB SPL, the ratio increased from 0.35 ± 0.02 to 2.43 ± 0.48 when the sound frequency went from 140 to 620 Hz (Fig. 2j and k). At 71 dB SPL, the ratio increased from 0.33 ± 0.02 to 2.42 ± 0.39. At 66 dB SPL, the ratio increased from 0.28 ± 0.02 to 1.57 ± 0.21. At 61 dB SPL, the ratio increased from 0.2 ± 0.02 to 1.22 ± 0.14. At 56 dB SPL, the ratio increased from 0.14 ± 0.02 to 1.03 ± 0.12. At 51 dB SPL, the ratio increased from 0.08 ± 0.02 to 0.76 ± 0.14.

To further characterize the SP-frequency relationship, the mean SP-to-AC amplitude ratio-frequency functions (Fig. 2k) were subjected to curve fitting analysis. Surprisingly, the ratio-frequency functions exhibited a 2-term exponential growth for all the sound intensities tested (red traces, Fig. 2l; see Supplemental Table 1 for the fit parameters). This is the first time such a relationship is established for low frequencies. The fact that the ratio-frequency functions exhibited an exponential growth suggests a gradual G_kf_ and G_ks_ tonotopicity for the 140–620 Hz range tested.

### The negative summating potentials characterize noise-induced hearing loss

To characterize SP polarity and frequency dependency in hearing loss conditions [36], the hearing organ in the temporal bone preparation described above was subjected to acoustic overstimulation consisting of pure tone of 140 Hz delivered at 98 dB SPL to induce a very mild temporary threshold shift [28, 36], after which the electrical potentials were recorded in response to the 160 sound stimulus combinations above.

**Fig. 3:**
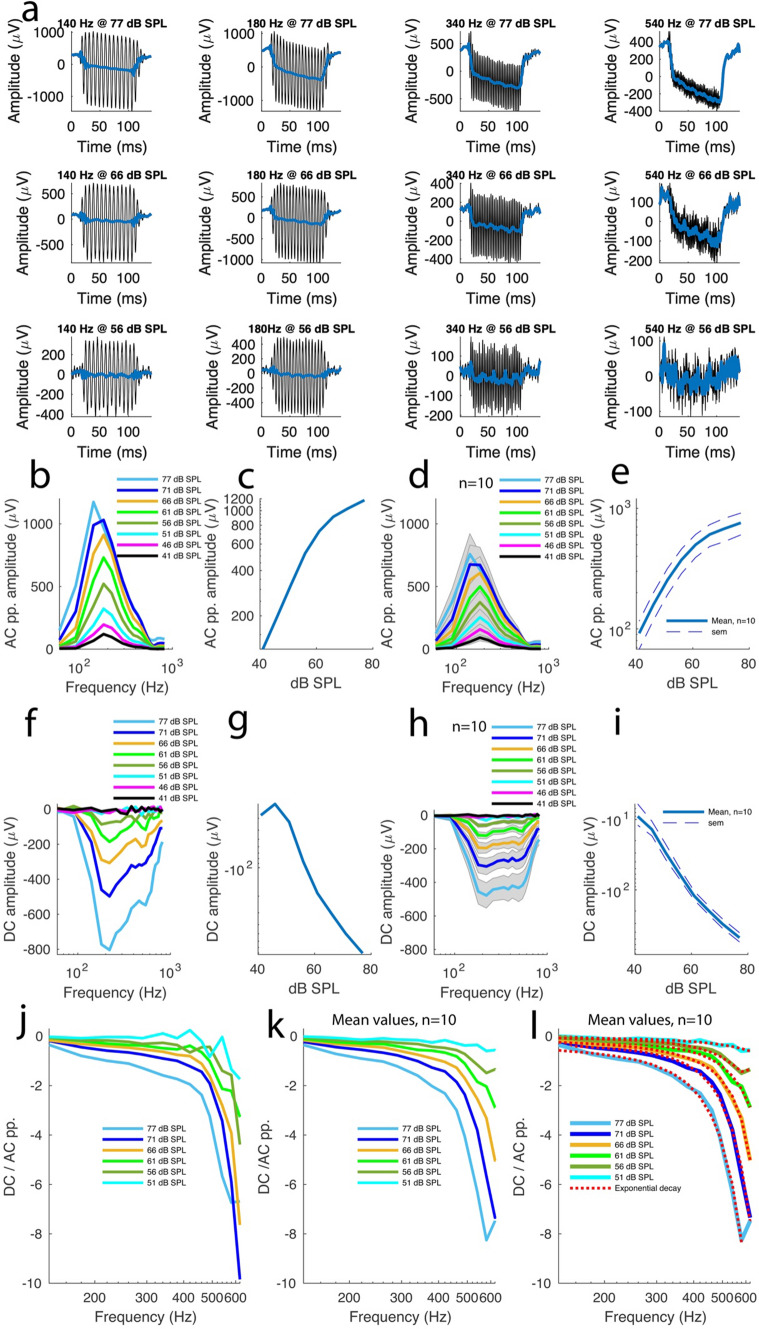
The negative summating potentials characterize noise-induced hearing loss. Acoustic overstimulation causes the SP to switch polarity to negative values. A-F, same description as in Fig. 2**A–F** after acoustic overstimulation at 98 dB SPL and 140 Hz (see Methods). Where indicated, n represents 10 preparations from 10 animals. **G** The nadir amplitudes for the SP tuning curves in F were plotted against sound intensity. **H** Averaged frequency-tuning curves for SP amplitudes across 10 preparations from 10 animals. **I** SP nadir amplitudes from the tuning curves in H plotted against sound intensity. **J** Ratios between SP nadir amplitude and AC peak-to-peak amplitude were plotted against frequency for different sound intensities for the preparation shown in (**A)**. **K** Same as in J for average data across 10 preparations from 10 animals. **L** The averaged plots in K were fitted with a double exponential function f(x) = f(x) = a*e^(b*x)^ + c*e^(d*x)^. See Supplemental Table 2 for the fitting parameter values. Where indicated, the shaded plots depict s.e.m

Figure 3a shows 12 of the 160 sound-evoked electrical responses recorded in the same preparation after exposure to acoustic trauma. The AC amplitudes were computed as described above and then plotted against frequency at different sound intensities. The tuning curves from this preparation show that AC amplitude remained frequency-tuned despite a decrease in amplitude (Figs. 3b vs. 2b). In addition, the maximum amplitudes retained a nonlinear relationship with sound intensity (Fig. 3c). Across 10 preparations from 10 guinea pigs, these features persisted. The peak-to-peak AC amplitudes were frequency-tuned with values of 758 ± 156 µV at 77 dB SPL; 675 ± 148 µV at 71 dB SPL; 607 ± 125 µV at 66 dB SPL; 500 ± 108 µV at 61 dB SPL; 375 ± 86 µV at 56 dB SPL; 254 ± 62 µV at 51 dB SPL; 158 ± 41 µV at 46 dB SPL and 92 ± 24 µV at 41 dB SPL (Fig. 3d). Plotted against the sound intensity, these AC amplitudes above retained a nonlinear relationship with the sound intensity with a slope of 20 ± 4 µV/dB SPL (Fig. 3e, n = 10 preparations from 10 guinea pigs).

To further characterize the noise-induced hearing loss described above, the AC peak amplitudes obtained in the control preparations and after exposure to acoustic overstimulation were statistically compared (Fig. 2e vs. Figure 3e), which revealed that the AC amplitude dependency in the ear health condition was significant (p < 0.0001, linear mixed modelling, LMM). In addition, the hearing loss across sound intensity was also statistically significant (p < 0.0001, LMM). Moreover, the combined effect of the sound intensity and ear health condition on the AC amplitude was significant (p < 0.0001, LMM).

To investigate the effect of the hearing loss on the SP, the SP waveforms were analysed in the 160 electrical recordings above (Fig. 3a). They exhibited a mirror image of their control counterparts, indicating that the noise-induced hearing loss caused the SP waveforms to switch polarity to negative values. In addition, the SP amplitudes had negative polarity across frequencies and sound intensities (Fig. 3f). The tuning curves revealed that the SP amplitude was frequency-tuned across sound intensities with nadir amplitude values of  – 804 µV at 77 dB SPL;  – 498 µV at 71 dB SPL;  – 307 µV at 66 dB SPL;  – 186 µV at 61 dB SPL;  – 86 µV at 56 dB SPL and  – 33 µV at 51 dB SPL. At 46 and 41 dB SPL, with nadir amplitude values of  – 21 and  – 27 µV respectively, the amplitudes lost their frequency-tuning as their values approached the baseline, a tendency already observed in control preparations above (Fig. 2f). Plotted against the sound intensities, the SP amplitudes from the noise-induced hearing loss preparations revealed a propensity to become more negative with the sound intensity (Fig. 3g).

Across 10 preparations from 10 different guinea pigs, the features above persisted (Fig. 3h). The SP nadir amplitude values of the averaged tuning curves were  – 478 ± 72 µV at 77 dB SPL;  – 305 ± 47 µV at 71 dB SPL;  – 196 ± 28 µV at 66 dB SPL; – 122 ± 18 µV at 61 dB SPL;  – 61 ± 11 µV at 56 dB SPL;  – 30 ± 7 µV at 51 dB SPL;  – 14 ± 3 µV at 46 dB SPL and   9 ± 3 µV at 41 dB SPL (Fig. 3h). Against frequency, these SP amplitudes confirmed that the SP became more negative with frequency with a slope of -12 ± 2 µV/dB SPL (n = 10, Fig. 3i), confirming that the negative SP polarity is the signature of noise-induced hearing loss.

Comparison of the SP peak amplitudes from the control preparations and the nadir amplitudes from noise-induced hearing loss preparations (Figs. 1i vs. 2i) revealed that the SP amplitude depended on the ear health condition (p < 0.0001, LMM) and that its dependency in sound intensity was significant (p < 0.0001, LMM). In addition, the combined effect of sound intensity and ear health condition on the SP amplitude was statistically significant (p < 0.0001, LMM).

To gain a deeper insight of the SP behaviour in the noise-induced hearing loss ears, the SP-to-AC amplitude ratios were plotted against frequencies using the same frequency band and intensities as for their control counterparts. In the example preparation shown Fig. 3j, the ratios became more negative with frequencies for all the sound intensities tested and this trend was confirmed across 10 preparations (Fig. 3k). Curve fitting revealed that the SP-to-AC amplitude ratio-frequency functions were characterised with a 2-term decreasing exponential function across the sound intensities tested (Fig. 3l; see Supplementary Table 2 for fit parameters), in agreement with the findings above that the negative SP is a signature of noise-induced hearing loss and that the 140–620 Hz range tested is characterised by a tonotopic basolateral K^+^ conductance.

### Frequency variation in the basolateral K^+^ channels

The SP-to-AC amplitude ratio functions above support that the cochlear region studied had frequency sensitive basolateral K^+^ conductance. Tonotopic variation along the cochlear spiral has been recently confirmed for the MET channels, which exhibited a large apex-to-base conductance gradient for the OHCs and a small one for the IHCs [37, 38]. If the basolateral K^+^ channels are also characterised by an apex-to-base conductance gradient, then they could have a conductance gradient, with a lower conductance towards 140 Hz and a higher conductance toward 620 Hz. If that is the case, then reducing the extracellular K^+^, which along with Ca^2+^ constitutes the majority of the MET currents [39–42], should affect more the higher frequency basolateral K^+^ conductance and the associated SP, thus confirming tonotopicity. A survey of the literature showed that application of 10–50 µM dihydrostreptomycin (DHS) abolishes MET current amplitudes of isolated hair cells while the Ca^2+^-driven adaptive components of the responses remained ostensibly prominent [43]. Because these adaptive components are Ca^2+^-specific, this suggests that DHS application can specifically block MET channel K^+^ entry.

**Fig. 4:**
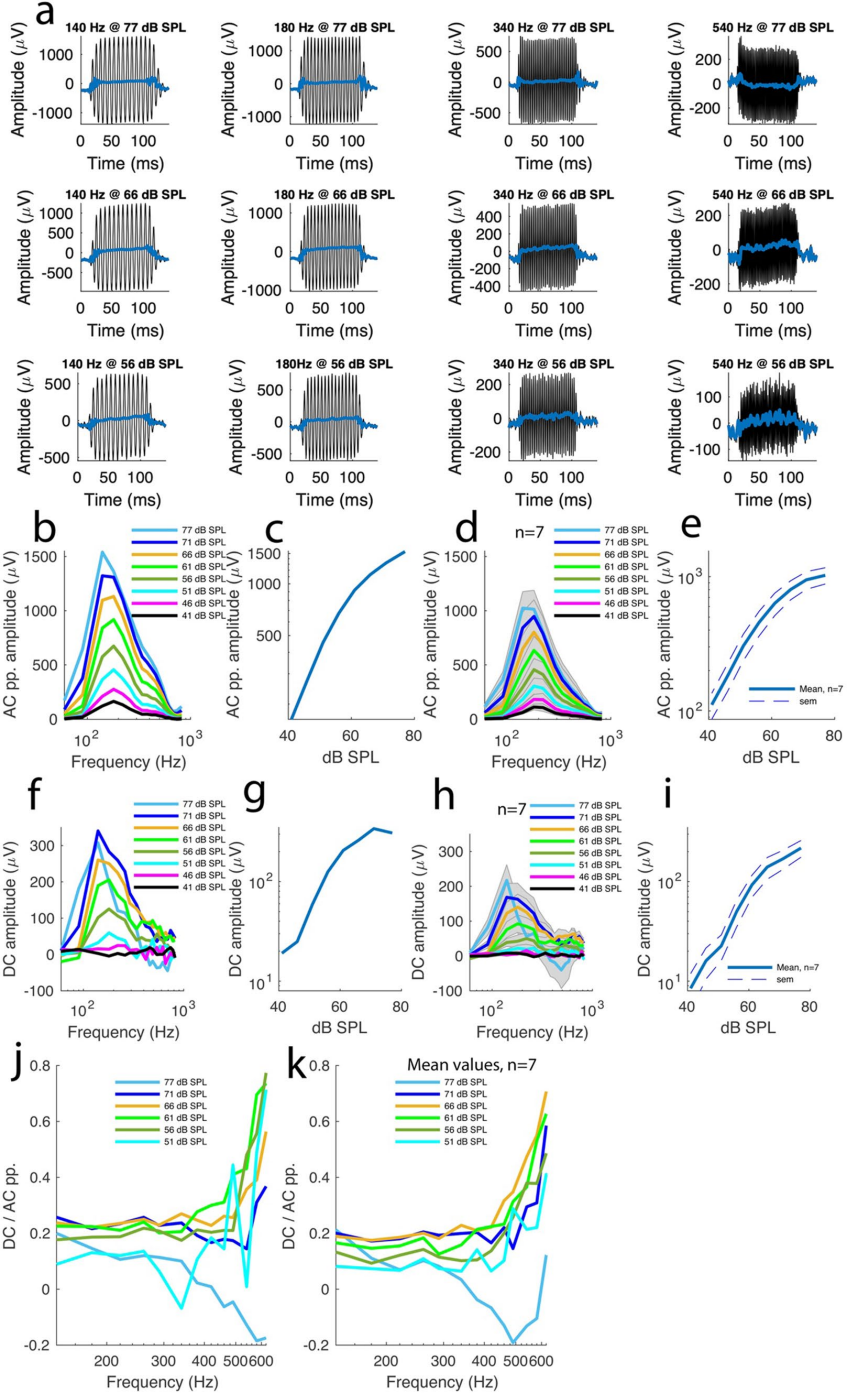
Tonotopic variation in the basolateral K^+^ channels. Dihydrostreptomycin (DHS, see Methods) application affected the higher frequency K^+^ conductance more than the lower frequency K^+^ conductance, revealing gradual conductance tonotopicity for the basolateral K^+^ channels. **A**-**K**, same description as in Fig. 2**A**–**K** after DHS application. Where indicated, n represents 7 preparations from 7 animals. Where indicated, shaded plots depict s.e.m

Figure 4a shows 12 of 160 electrical recordings obtained in the same preparation at different frequencies and sound intensities after application of micromolar concentration of DHS with the recording glass electrode in the endolymphatic space (see Methods). Frequency tuning curves from this preparation revealed a robust but substantially reduced AC response amplitude relative to the control preparations, suggesting that DHS was effective at reducing K^+^ currents. However, the AC amplitude remained frequency-tuned, with peak amplitudes of 1540 µV at 77 dB SPL; 1322 µV at 71 dB SPL; 1131 µV at 66 dB SPL; 918 µV at 61 dB SPL; 674 µV at 56 dB SPL; 455 µV at 51 dB SPL; 275 µV at 46 dB SPL; 167 µV at 41 dB SPL (Fig. 4b). Against sound intensity, these AC amplitudes from this preparation exhibited a nonlinear relationship (Fig. 4c). Across 7 preparations, the finding above were confirmed. As shown by Fig. 4d, the AC amplitudes were frequency-tuned, and the peak amplitudes had values of 1023 ± 141 µV at 77 dB SPL; 947 ± 142 µV at 71 dB SPL; 802 ± 129 µV at 66 dB SPL; 634 ± 120 µV at 61 dB SPL; 456 ± 95 µV at 56 dB SPL; 305 ± 68 µV at 51 dB SPL; 182 ± 42 at 46 dB SPL and 111 ± 25 µV at 41 dB SPL. Plotted against the sound intensity, these mean AC amplitudes exhibited nonlinearity despite the reduced amplitudes (Fig. 4e). Compared to the control preparations, the sound intensity dependency of the AC amplitude in presence of DHS was statistically significant (p < 0.0001, LMM) and the DHS-induced decrease in AC amplitude was also statically significant (Figs. 2e vs 4e, p < 0.0001, LMM). In addition, the combined effect of sound intensity and DHS was also significant (p < 0.0001, LMM). These data indicate that DHS induced a moderate but significant effect on the currents passing through the MET channels.

To investigate the effect of K^+^ current reduction on the SP response, the SP amplitudes were systematically analysed in the electrical potential recordings obtained in the 160 recordings above (Fig. 4a). The recordings revealed that several SP waveforms exhibited a control-like shape albeit with a smaller amplitude while a small number of them exhibited negative polarity especially at high frequencies and sound intensities (Fig. 4a).

Overall, the SP peak amplitudes had values of 308 µV at 77 dB SPL; 340 µV at 71 dB SPL; 260 µV at 66 dB SPL; 206 µV at 61 dB SPL; 125 µV at 56 dB SPL; 59 µV at 51 dB SPL; 24 µV at 46 dB SPL and 19 µV at 41 dB SPL (Fig. 4f). The amplitude-sound intensity relationship was nonlinear (Fig. 4g).

Averaging the tuning curves across 7 preparations (Fig. 4h) confirmed the findings above. First, the SP amplitude was markedly reduced across frequencies with mean peak values of 217 ± 41 µV at 77 dB SL; 169 ± 37 µV at 71 dB SPL; 140 ± 34 µV at 66 dB SPL; 93 ± 23 µV at 61 dB SPL; 50 ± 15 µV at 56 dB SPL; 23 ± 6 µV at 51 dB SPL; 16 ± 6 µV at 46 dB SPL, 8 ± 3 µV at 41 dB SPL and the relationship between the amplitudes and sound intensity was nonlinear (Fig. 4i). Second, the tuning curves revealed that a switch in SP polarity to negative values occurred at around 340 Hz (Fig. 4h) with a nadir amplitude value of  – 40 ± 53 µV at 77 dB SPL. These data confirm the tonotopicity of the basolateral K^+^ conductance.

Statistically speaking, the dependence of the SP amplitude in the sound intensity in DHS-treated preparations was significant (p < 0.0001, LMM). In addition, the DHS-induced decrease in SP amplitude relative to control data was significant (Figs. 2i vs 4i, p < 0.0001, LMM) and the combined effect of sound intensity and DHS on the SP amplitude was also significant (p < 0.0001, LMM).

Plotting the SP-to-AC amplitude ratios against frequencies, using the same frequency range used for the control data, confirmed the SP amplitude decrease in the frequency domain above 340 Hz. In the example preparation shown in Fig. 4a, at 77 dB SPL, the ratios were positive up to 340 Hz and grew negative beyond this frequency (Fig. 4j).

For the 51–71 dB SPL intensity range, the ratios lost their rapid growth-exponential component, with values ranging between 0.37 and 0.77 at 620 Hz (Fig. 4j). These findings were confirmed across 7 preparations (Fig. 4k) where the mean ratios ranged between 0.41 and 0.71 at 620 Hz for the 51–71 dB SPL sound intensity range, a decrease from their control counterparts that had a value range between 0.66 and 1.85.

Statistically speaking, the DHS-induced change in the SP-to-AC amplitude ratios was significant relative to the control conditions (p < 0.0001, generalized linear modelling).

In summary the data support that conductance tonotopicity for the basolateral K^+^ channels underlies SP production along the frequency axis.

### Does the SP polarity depend solely on extracellular potassium?

**Fig. 5:**
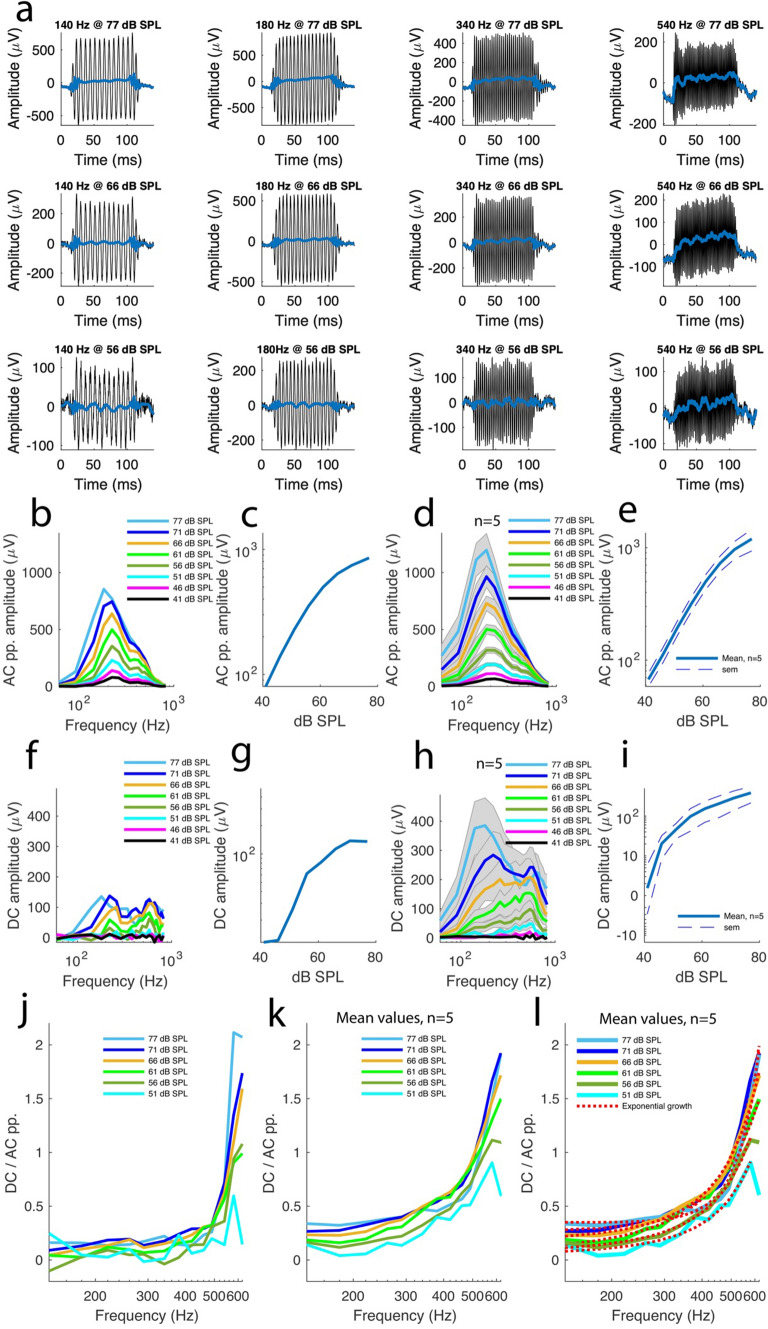
Effect of decreasing extracellular calcium on the summating potentials. Decreasing extracellular calcium with EGTA similarly affected the AC and SP amplitude, supporting the idea that the SP amplitude and shape are controlled by extracellular K^+^. **A**–**L**, same description as in Fig. 2**A**–**L** after EGTA application. Where indicated, n represents 5 preparations from 5 animals. Shaded plots depict s.e.m. The averaged plots in** K** were fitted with a double exponential function $f\left( x \right)\, = \,a*e^{(b*x)} \, + \,c*e^{(d*x)}$ for all sound intensities except for 51 dB SPL, where the equation $f\left( x \right)\, = \,a*e^{(bx)}$ was used. See Supplemental Table 3 for the fitting parameter values

We showed previously that reducing extracellular calcium with the application of the Ca^2+^ chelator EGTA (ethylene glycol-bis(β-aminoethyl ether)-N,N,N′,N′-tetraacetic acid) affects the cochlear microphonic amplitudes at 64 dB SPL [36]. However, the effect on the amplitudes at other sound intensities has not been investigated. Additionally, it is currently unclear how EGTA application affects the SP waveforms and the relationship between SP and AC amplitudes. To address this issue, electrical potential recordings in response to the 160 stimulus combinations described above were acquired after micromolar EGTA application (see Methods). Figure 5a shows 12 of 160 recordings from the same preparation at different frequencies and sound intensities after EGTA application. The AC amplitudes from this preparation were evidently reduced relative to the control data but remained frequency-tuned (Fig. 5b) with peak values of 853 µV at 77 dB SPL; 745 µV at 71 dB SPL; 638 µV at 66 dB SPL; 499 µV at 61 dB SPL; 354 µV at 56 dB SPL; 229 µV at 51 dB SPL; 140 µV at 46 dB SPL and 80 µV at 41 dB SPL. In addition, these amplitudes depended nonlinearly in sound intensity (Fig. 5c).

Across 5 preparations, the features above persisted. The amplitudes were evidently smaller compared to control preparation but remained frequency-tuned (Fig. 5d) with peak amplitude values of 1197 ± 261 µV at 77 dB SPL; 964 ± 172 µV at 71 dB SPL; 729 ± 114 µV at 66 dB SPL; 502 ± 76 µV at 61 dB SPL; 319 ± 48 µV at 56 dB SPL; 192 ± 28 µV at 51 dB SPL; 111 ± 17 µV at 46 dB SPL and 67 ± 9 µV at 41 dB SPL (n = 5 preparations from 5 different animals). These mean peak amplitudes had a nonlinear dependency on the sound intensity with a slope of 33 ± 8 µV/dB SPL (Fig. 5e, n = 5 preparations from 5 different animals).

Compared to the control conditions (Fig. 2), the EGTA-induced decrease in AC amplitude was significant (p < 0.001, LMM), the slope difference was significant (p < 0.0001, LMM) and the combined effect of sound intensity and EGTA was also significant (p < 0.0001).

To investigate the effect of EGTA application on the SP response, the SP waveforms were analysed in each of the 160 recordings obtained in each preparation after EGTA application. In the example preparation above, the SP amplitude was markedly reduced compared to the control data and the frequency-tuning curves exhibited a double peak (Fig. 5f). The amplitudes of the largest peak were 135 µV at 77 dB SPL; 138 µV at 71 dB SPL; 113 µV at 66 dB SPL; 82 µV at 61 dB SPL; 62 µV at 56 dB SPL; 26 µV at 51 dB SPL; 12 µV at 46 dB SPL and 12 µV at 41 dB SPL. These peak amplitudes exhibited a nonlinear dependence on the sound intensity (Fig. 5g). However, in this case, the saturation at high SPLs was more pronounced compared to control conditions and is consistent with calcium role in setting the dynamic range of the MET channels [40–42].

Across 5 preparations, most of the features above persisted. The double-peak tuning persisted at sound intensity levels ≥ 61 dB SPL but at low SPLs the two peaks merged progressively as the sound intensity decreased. Moreover, the SP amplitude saturation also persisted and was prominent at 580 Hz where the amplitudes at 77, 71 and 66 dB SPLs had a similar amplitude value (Fig. 5h). Overall, the peak amplitude values were 385 ± 170 µV at 77 dB SPL; 284 ± 144 µV at 71 dB SPL; 208 ± 115 µV at 66 dB SPL; 154 ± 84 µV at 61 dB SPL; 98 ± 56 µV at 56 dB SPL; 46 ± 19 µV at 51 dB SPL; 20 ± 12 µV at 46 dB SPL and 2 ± 4 µV at 41 dB SPL. These amplitudes had a nonlinear dependence on the sound intensity with a slope of 11 ± 6 µV/dB SPL while also exhibiting a pronounced saturation at higher sound intensity (Fig. 5i), confirming the role of calcium in setting the hearing dynamic range as argued above.

Statistically speaking, the EGTA-induced SP amplitude decrease, the slope difference, and the combined effect of frequency and EGTA on the amplitude were significant relative to their control counterparts with p < 0.01, p < 0.0001 and p < 0.001 respectively (LMM).

To further characterize the effect of calcium extracellular removal on the SP, the SP-to-AC amplitude ratios were computed for the frequency band and sound intensity range used for their control counterparts.

In the example preparation shown in Fig. 5a, the ratios exhibited a control-like behaviour i.e., increased with frequency irrespective of the sound intensity tested (Fig. 5j) and this trend persisted across 5 preparations (Fig. 5k). Curve-fitting the mean ratio data from the 5 preparations revealed that the ratio-frequency relationship was a 2-term exponential growth for the sound intensities studied except for the 51 dB SPL where a single growth exponential function was used (red trace, Fig. 5l; see Supplemental Table 3 for the fitting parameters). Compared to the control conditions, the ratios did not exhibit a statistically significant difference relative to their control counterparts (for example, p = 0.09 at 77 dB SPL, generalized linear modelling), indicating that decreasing extracellular calcium affected the AC and SP amplitude similarly, which supports the idea that the SP amplitude and shape are controlled by extracellular K^+^[12].

## Discussion

**Fig. 6:**
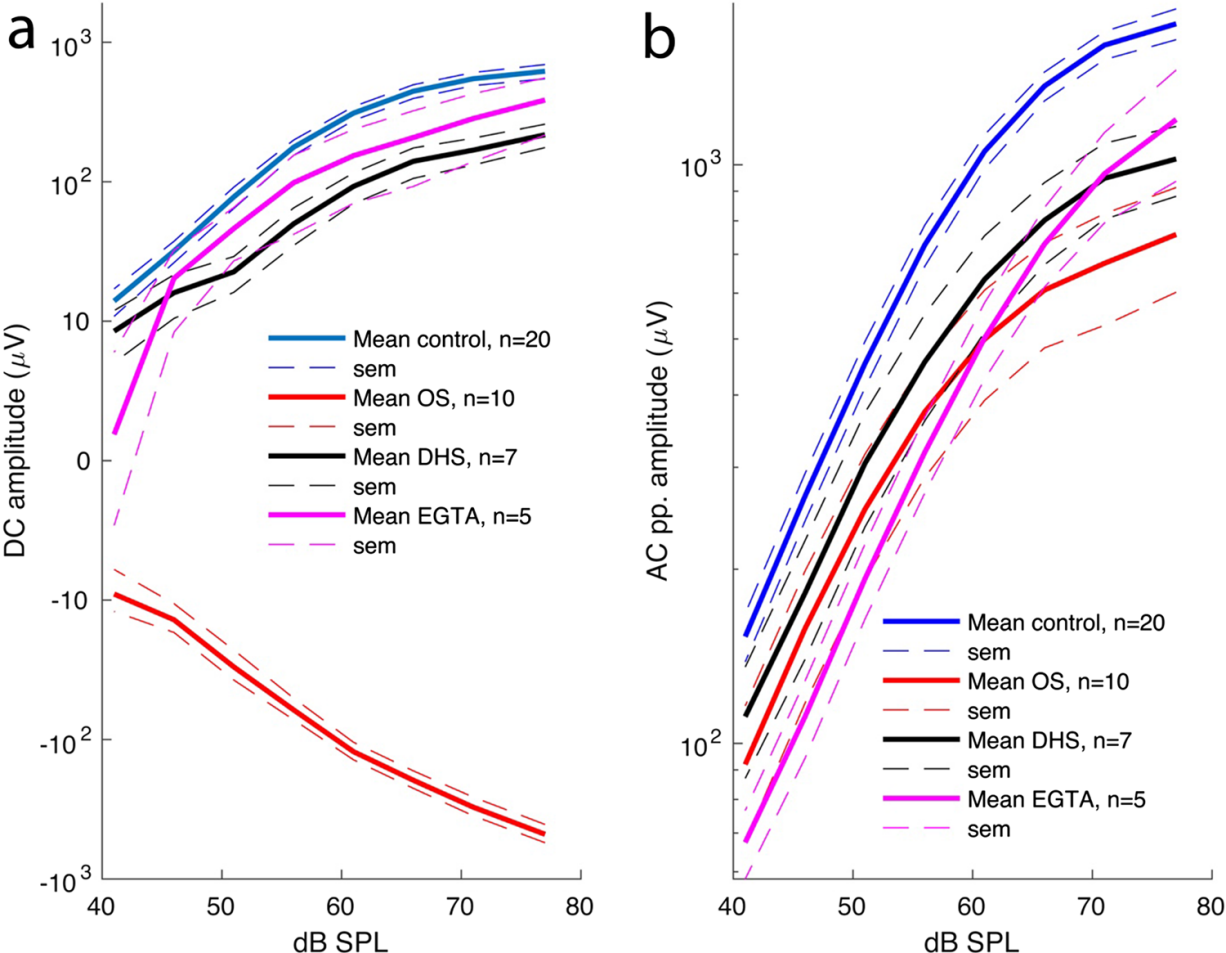
Summary plots highlighting the effects of various treatments on the SP (**A**) and AC (**B**) responses

Since Davis and colleagues first described the SP seven decades ago [20], its exact polarity and physiological roles have remained elusive. As demonstrated in this study, the SP encodes the ear health status (see summary plots in Fig. 6). In normal ears, the SP is positive and relative to the AC response, its amplitude grows exponentially with frequency, while in a hearing-loss ears, the SP is negative and its amplitude relative to the AC response decreases exponentially with frequency. These findings are in contrast to previous studies that reported both positive and negative SPs in normally hearing ears [25, 26]. The only case where bipolar SPs were obtained in the present study consisted in a pathological situation in which MET channels were partially blocked with DHS, suggesting a change in the operating point of the hair cells. However, these DHS experiments made it possible to establish that a tonotopic variation of basolateral K^+^ channels exists in the cochlear apex as described above, possibly due to stoichiometric changes in the channel assembly established for MET channels [37].

To address the mechanisms underlying SP polarity change following acoustic overstimulation, attention was paid to a recent study that attempted to dissect the SP into hair cell and neural components using pharmacological manipulations and electrical recordings in both control animals and animals with neutralized OHCs and neural terminals [44]. According to this study, the IHCs exhibit a negative SP, while the OHCs exhibit a positive SP. Such an idea is tempting because recent OHC vibrometry measurements from the Oghalai group have exhibited a positive DC polarity [45]. However, as argued above, with a small resting open probability for the IHCs and a 50% one for the OHCs, the IHCs should be the main source of SP while the OHCs contribute predominantly to the AC response [15]. Thus, the tonotopic variation in G_Kf_ and G_Ks_ established by the DHS experiments could apply only to the IHCs. It is noteworthy from a physiological standpoint that the sources of the cochlear microphonic components have a specific tonotopy, with the IHCs having a tonotopicity for the SP-producing channels and the OHCs having a tonotopic variation for the AC response-producing channels. Thus, the shift in SP polarity observed in the present study after acoustic overstimulation may reflects a change in the operating point of the hair cells. In fact, past SP measurements from the 90 s in the guinea pig temporal bone showed that application of small amount of positive and negative pressure caused the SP polarity to switch accordingly [46]. These findings imply that the noise-induced polarity switch observed in the present study reflect a change in the operating point of the hair cells as argued above. Although such a change could affect both the OHCs and the IHCs, the IHCs are likely to remain the main SP source because the OHCs don’t appear to be equipped with the SP producing apparatus such as the basolateral K^+^ channels found in the IHCs [12].

From an engineering point of view, using the SP polarity to differentiate between normal and injured ears could potentially facilitate the brain's ability to decode faint sounds by activating the OHC cochlear amplifier via the olivocochlear efferents [47]. Although a negative SP has been suggested to be characteristic of Menière’s disease [46], it is generally challenging to isolate the SP signal in electrocochleograms because its peak is defined differently from one study to another [20, 44, 48, 49]. Furthermore, it typically requires sound intensities approaching 120 dB SPL [50], which can cause permanent hearing loss [51–53]. These factors make it challenging to use the SP signal for reliable clinical diagnosis of hearing and balance dysfunction [48].Nevertheless, these obstacles could be mitigated with innovative data processing techniques that seem to simplify the isolation of the SP from neural components in human electrocochleograms [54].

This study also tested and confirmed the idea that the SP production is controlled by potassium signaling [12]. A previous attempt to block cochlear potassium channels in vivo with 4-aminopyridine was not conclusive [49]. This was not surprising given the known severe side effects of 4-aminopyridine for the cochlea, and the fact that this drug was used at 5–30 mM range supports its poor specificity. By contrast, DHS used in the present study to block potassium currents was highly specific because a substantial effect was achieved at micromolar levels (see Methods).

In addition to confirming the importance of potassium in SP production, the experiments above constitute a mechanistic basis for understanding how noise-induced hearing loss develops. These findings may inspire further research that could utilize the SP polarity to diagnose noise-induced hearing loss, a challenge that is currently hindered by uncertainty surrounding the SP polarity.

Overall, the findings above suggest that the SP polarity can serve as a reliable indicator of ear health status, and that modulating the operating point of hair cells via potassium signaling could potentially be a therapeutic target for preventing noise-induced hearing loss.

## Materials and methods

### The temporal bone preparation from the guinea pig

This study complied with all relevant ethical regulations for animal testing and research. All the animal procedures described in this study were approved by the Regional Ethics Committee in Linköping, Sweden (Permit number DNR 5111–2019).

Guinea pigs of either sex and aged between 2 and 5 weeks were anesthetized with an intraperitoneal injection of sodium pentobarbital (0.8 mL, 50 mg/mL), decapitated and then the temporal bone was isolated before it was mounted onto a custom chamber holder. Immediately after, the bulla was gently opened to expose the cochlear base and apex; and then the cochlea and middle ear were immersed into oxygenated cell culture medium (minimum essential medium with Earle’s balanced salts, room temperature) after which two small openings were made at the cochlear base and apex respectively under dissection microscopy. It is the apical opening that is used for the measurements reported in the present study.

Using a fine plastic tube connected into the basal opening, oxygenated cell culture medium (same as above) was allowed to perfuse gently through the cochlea and exit through the apical opening at the rate of 0.6 ml/h from an external 4 ml-tank solidly mounted on a stand to ensure that this position was fixed across experiments. This perfusion system keeps the preparation alive for up to 4 h [32]. The details underlying these surgical procedures have been recently detailed elsewhere [28]. Given that the preparation is typically readied for experimentation within an hour following decapitation, the aforementioned survival period provides ample time for conducting the experiments in stable conditions. Because of the custom chamber design, the ear canal was protected from the fluid, thus making it possible to acoustically stimulate the hearing organ with a calibrated speaker inserted into the ear canal. However, the fluid immersion of the middle ear is known to attenuate the sound stimulus by at least 20 dB SPL [28, 55]. Consequently, the sound intensity values given throughout the text have been corrected accordingly. Since the preparation rests on a rotative mount, the apical opening is oriented such that the hearing organ is visualized in the cross-section orientation by reflection confocal microscopy, an imaging technique that does not require sample staining or any other form of treatment [28]. This orientation makes it easy to perpendicularly insert a thin glass electrode through an otherwise intact Reissner’s membrane closer and parallel to the stria vascularis with a computer-controlled micromanipulator. The glass electrode above is used to extracellularly record the sound-evoked electrical responses of the hair cells. Since the recording setup was optimized to work with the left ears, only one ear per animal was used and therefore the number of preparations is the same as the number of animals throughout the text.

### Electrophysiological recordings

To prepare for the electrode above, a thin capillary glass (World Precision Instruments) was pulled with a standard puller and filled with an artificial endolymph solution containing 1.3 mM NaCl, 31 mM KHCO_3_, 128.3 mM KCl, and 0.023 mM CaCl_2_ (pH 7.4, osmolality 300 mOs/kg) and then beveled at an angle of 30° until an impedance of 2–3 MΩ was reached. To record the sound-evoked electrical responses of the hair cells mentioned above, a Dagan Ix1 amplifier (Dagan Instruments) and custom LabView software were used.

Where indicated, either EGTA (100 µM) or dihydrostreptomycin (DHS, up to 100 µM) were added to the electrode solution before the bevelling step above. Drug delivery in the endolymphatic space where the stereocilia reside, was achieved by application of low pressure (≤ 4 psi) for a few seconds at the back end of the electrode with a Picospritzer. This drug delivery approach is highly precise because the volume injected is limited by the pressure and the tiny opening of the bevelled electrode. Therefore drug delivery only takes place once the pressure has been applied. Typically, under the injection parameters above, only a single and tiny droplet is released from the electrode, meaning that the effective drug concentration in the endolymphatic space after delivery is much lower than in the electrode once the droplet is allowed to diffuse, typically within minutes of injection (for instance, injection of stereocilia-staining dyes with this method under confocal microscopy monitoring achieves its effect nearly instantly).

With previous reports on the summating potential (SP) instability in mind and guided by many years of observing the SP behaviour in our preparations, care was taken to ensure that the preparation SP has stabilized before the measurements presented in this study could be performed. In fact, the LabView acquisition software has been optimized for this very purpose. Once a series of sound-evoked electrical responses are acquired at a given sound intensity (typically 20 frequencies equally distributed between 60 and 820 Hz at around 60 dB SPL), the raw data are automatically processed, and the AC and SP tuning curves displayed on the acquisition screen. In addition, plots corresponding to successive acquisition are continuously assigned a different colour code and superimposed. This makes it possible for the user to continuously assess the stability of both the AC and SP tuning curves over time. Once both peaks have stabilized for at least 8–15 min, the preparation is deemed stable for SP investigations, during which the acquisition of the electrical recordings for the 20 frequencies above is repeated for 8 different sound intensities, ranging from 41 to 77 dB SPL and the files saved for offline data processing in MATLAB (The MathWorks).

Where indicated, acoustic overstimulation was performed at 98 dB SPL and 140 Hz, a frequency slightly below the best frequency of the recording location (180 Hz), as previously described [36] except that the duration of the exposure was reduced to about one minute.

### Statistical analysis

In this study, amplitude – sound intensity functions as well as SP-to-AC amplitude ratios – frequency functions were statistically compared between different preparation types (control, noise-induced hearing loss preparations, or preparations treated with pharmacological substances; the number of animals per experiment type is given throughout the Results section and Figure legends, where indicated). However, because of the repetitive nature of the sampling (for example, amplitudes are measured at several frequencies and intensities in the same preparation, correlations arise inevitably and consequently have to be dealt with by linear mixed modelling (LMM) [28]. In the model, the random effect was the preparation ID, whereas the fixed effect was frequency or sound intensity, and the preparation type. The dependent variable was the amplitude or ratio, where indicated. The calculations were done using the lme4 and nlme packages in RStudio.

Curve fitting for the SP-to-AC amplitude ratio-frequency functions was performed in MATLAB (The MathWorks). After test-fitting the data with different function types, the exponential functions agreed best with the experimental data. The fitting parameters are given in the Supplemental Tables, where indicated.

### Supplementary Information

Below is the link to the electronic supplementary material.

Supplementary file1 (DOCX 17 KB)

## Data Availability

All data supporting the findings from this study are available from the corresponding author upon reasonable request.

## References

[1] Assad JA (1991). Tip-link integrity and mechanical transduction in vertebrate hair cells. Neuron.

[2] Holton T, Hudspeth AJ (1986). The transduction channel of hair cells from the bull-frog characterized by noise analysis. J Physiol.

[3] Crawford AC (1989). Activation and adaptation of transducer currents in turtle hair cells. J Physiol.

[4] Tan X (2013). Electrical tuning and transduction in short hair cells of the chicken auditory papilla. J Neurophysiol.

[5] Beurg M (2006). A large-conductance calcium-selective mechanotransducer channel in mammalian cochlear hair cells. J Neurosci Off J Soc Neurosci.

[6] Géléoc GS (1997). A quantitative comparison of mechanoelectrical transduction in vestibular and auditory hair cells of neonatal mice. Proc Biol Sci.

[7] He DZZ (2004). Mechanoelectrical transduction of adult outer hair cells studied in a gerbil hemicochlea. Nature.

[8] Jia S (2007). Mechanoelectric transduction of adult inner hair cells. J Neurosci Off J Soc Neurosci.

[9] Kennedy HJ (2003). Fast adaptation of mechanoelectrical transducer channels in mammalian cochlear hair cells. Nat Neurosci.

[10] Kros CJ (1992). Mechano-electrical transducer currents in hair cells of the cultured neonatal mouse cochlea. Proc R Soc Lond Ser B.

[11] Dillard LK (2022). Prevalence and global estimates of unsafe listening practices in adolescents and young adults: a systematic review and meta-analysis. BMJ Glob Health.

[12] Fettiplace R (2017). Hair cell transduction, tuning and synaptic transmission in the mammalian cochlea. Compr Physiol.

[13] Marcotti W (2003). Developmental changes in the expression of potassium currents of embryonic, neonatal and mature mouse inner hair cells. J Physiol.

[14] Kimitsuki T (2003). Potassium current properties in apical and basal inner hair cells from guinea-pig cochlea. Hear Res.

[15] Johnson SL (2011). Prestin-driven cochlear amplification is not limited by the outer hair cell membrane time constant. Neuron.

[16] Mammano F, Ashmore JF (1996). Differential expression of outer hair cell potassium currents in the isolated cochlea of the guinea-pig. J Physiol.

[17] Kros CJ, Crawford AC (1990). Potassium currents in inner hair cells isolated from the guinea-pig cochlea. J Physiol.

[18] Kros CJ (2007). How to build an inner hair cell: challenges for regeneration. Hear Res.

[19] Marcotti W, Kros CJ (1999). Developmental expression of the potassium current IK, n contributes to maturation of mouse outer hair cells. J Physiol.

[20] Davis H (1950). The excitatory process in the cochlea*. Proc Natl Acad Sci.

[21] Davis H (1958). Summating potentials of the cochlea. Am J Physiol-Leg Content.

[22] Pierson M, Dallos P (1976). Re-examination of avian cochlear potentials. Nature.

[23] Dallos P (1982). Intracellular recordings from cochlear outer hair cells. Science.

[24] Russell IJ, Sellick PM (1983). Low-frequency characteristics of intracellularly recorded receptor potentials in guinea-pig cochlear hair cells. J Physiol.

[25] Cheatham MA (2004). Cochlear function in Prestin knockout mice. J Physiol.

[26] Dallos P (1970). Cochlear summating potentials: composition. Science.

[27] Cody AR, Russell IJ (1987). The response of hair cells in the basal turn of the guinea-pig cochlea to tones. J Physiol.

[28] Hakizimana P, Fridberger A (2021). Inner hair cell stereocilia are embedded in the tectorial membrane. Nat Commun.

[29] Ulfendahl M (1989). Isolated cochlea preparation for the study of cellular vibrations and motility. Acta Oto-Laryngol Suppl.

[30] Hakizimana P (2012). Sound-induced length changes in outer hair cell stereocilia. Nat Commun.

[31] Brown MC (1983). Cochlear inner hair cells: effects of transient asphyxia on intracellular potentials. Hear Res.

[32] Brownell WE (2011). Membrane cholesterol modulates cochlear electromechanics. Pflugers Arch.

[33] Tomo I (2007). Imaging the living inner ear using intravital confocal microscopy. Neuroimage.

[34] Honrubia V, Ward PH (1968). Longitudinal distribution of the cochlear microphonics inside the cochlear duct (guinea pig). J Acoust Soc Am.

[35] Burwood G (2022). Best frequencies and temporal delays are similar across the low-frequency regions of the guinea pig cochlea. Sci Adv.

[36] Strimbu CE (2019). Control of hearing sensitivity by tectorial membrane calcium. Proc Natl Acad Sci U S A.

[37] Beurg M (2018). Variable number of TMC1-dependent mechanotransducer channels underlie tonotopic conductance gradients in the cochlea. Nat Commun.

[38] Beurg M (2015). Subunit determination of the conductance of hair-cell mechanotransducer channels. Proc Natl Acad Sci.

[39] Corey DP, Hudspeth AJ (1979). Ionic basis of the receptor potential in a vertebrate hair cell. Nature.

[40] Peng AW (2013). Adaptation of mammalian auditory hair cell mechanotransduction is independent of calcium entry. Neuron.

[41] Caprara GA (2020). Decades-old model of slow adaptation in sensory hair cells is not supported in mammals. Sci Adv.

[42] Corns LF (2014). Calcium entry into stereocilia drives adaptation of the mechanoelectrical transducer current of mammalian cochlear hair cells. Proc Natl Acad Sci.

[43] Ricci A (2002). Differences in mechano-transducer channel kinetics underlie tonotopic distribution of fast adaptation in auditory hair cells. J Neurophysiol.

[44] Pappa AK (2019). Hair cell and neural contributions to the cochlear summating potential. J Neurophysiol.

[45] Dewey JB (2021). Cochlear outer hair cell electromotility enhances organ of Corti motion on a cycle-by-cycle basis at high frequencies in vivo. Proc Natl Acad Sci.

[46] Fridberger A (1997). Pressure-induced basilar membrane position shifts and the stimulus-evoked potentials in the low-frequency region of the guinea pig cochlea. Acta Physiol Scand.

[47] Rabbitt RD (2009). Power Efficiency of Outer Hair Cell Somatic Electromotility. PLOS Comput Biol.

[48] Eggermont JJ (2017) Ups and downs in 75 years of electrocochleography. Front Syst Neurosci 11. Available at: https://www.frontiersin.org/articles/10.3389/fnsys.2017.00002. Accessed November 13, 2022 10.3389/fnsys.2017.00002 PMC5259695 28174524

[49] van Emst MG (1996). 4-Aminopyridine effects on summating potentials in the guinea pig. Hear Res.

[50] Santarelli R (2009). Abnormal cochlear potentials from deaf patients with mutations in the otoferlin gene. J Assoc Res Otolaryngol.

[51] Bohne BA, Rabbitt KD (1983). Holes in the reticular lamina after noise exposure: implication for continuing damage in the organ of Corti. Hear Res.

[52] Hirose K, Liberman MC (2003). Lateral wall histopathology and endocochlear potential in the noise-damaged mouse cochlea. J Assoc Res Otolaryngol JARO.

[53] Wang Y (2002). Dynamics of noise-induced cellular injury and repair in the mouse cochlea. J Assoc Res Otolaryngol JARO.

[54] Vasilkov V (2023). Isolating auditory-nerve contributions to electrocochleography by high-pass filtering: A better biomarker for cochlear nerve degeneration?. JASA Express Lett.

[55] Ulfendahl M (1996). Mechanical response characteristics of the hearing organ in the low-frequency regions of the cochlea. J Neurophysiol.

