# Supplementary material for: The summating potential polarity encodes the ear health condition
Source: Cell Mol Life Sci. 2023 May 24;80(6):163. doi: 10.1007/s00018-023-04809-5 (PMC10209229; doi:10.1007/s00018-023-04809-5)
Supplement: Supplementary file 1 — Supplementary file1 (DOCX 17 KB) [file 18_2023_4809_MOESM1_ESM.docx]

**Supplemental Table 1. Fitting parameters for Figure 2L**

| **Sound Intensity (dB SPL)** | **a** | **b** | **c** | **d** | **R²** |
| --- | --- | --- | --- | --- | --- |
| 77 | 0.30 | 0.0011 | 2.08 x 10⁻⁵ | 0.018 | 1.0 |
| 71 | 0.27 | 0.0016 | 8.60 x 10⁻⁶ | 0.019 | 1.0 |
| 66 | 0.24 | 0.0018 | 3.31 x 10⁻⁵ | 0.016 | 1.0 |
| 61 | 0.20 | 0.0017 | 5.39 x 10⁻⁴ | 0.011 | 1.0 |
| 56 | 0.15 | 0.0019 | 5.70 x 10⁻⁴ | 0.011 | 1.0 |
| 51 | 0.02 | 0.0055 | 9.37 x 10⁻² | -0.00020 | 0.98 |

**Supplemental Table 2. Fitting parameters for Figure 3L**

| **Sound Intensity (dB SPL)** | **a** | **b** | **c** | **d** | **R²** |
| --- | --- | --- | --- | --- | --- |
| 77 | -0.32 | 0.0034 | -0.014 | 0.010 | 1.00 |
| 71 | -0.26 | 0.0012 | -0.022 | 0.0093 | 1.00 |
| 66 | -0.12 | 0.0051 | -4.7 x 10⁻⁵ | 0.017 | 1.00 |
| 61 | -0.10 | 0.0026 | -0.011 | 0.0086 | 0.99 |
| 56 | -0.030 | 0.0067 | 2.4 x 10⁻¹⁷ | 0.061 | 0.99 |
| 51 | 174 | -0.058 | -0.036 | 0.0045 | 0.93 |

**Supplemental Table 3. Fitting parameters for Figure 5L**

| **Sound Intensity (dB SPL)** | **a** | **b** | **c** | **d** | **R²** |
| --- | --- | --- | --- | --- | --- |
| 77 | 0.36 | -5.8 x 10⁻⁴ | 0.0038 | 0.0098 | 0.99 |
| 71 | 0.024 | 0.0070 | 0.24 | -7.0 x 10⁻⁴ | 0.99 |
| 66 | 0.099 | -9.0 x 10⁻⁴ | 0.063 | 0.0053 | 1.00 |
| 61 | -7.3 | 0.0029 | 7.3 | 0.0029 | 1.00 |
| 56 | 5199 | 0.0023 | -5199 | 0.0023 | 0.98 |
| 51 | 0.067 | 0.0041 | N/A | N/A | 0.83 |
